# Exploration of the Wild Edible Plants Used for Basic Health Care by Local People of Bahawalpur and Adjacent Regions, Pakistan

**DOI:** 10.3390/foods12193557

**Published:** 2023-09-25

**Authors:** Tauseef Anwar, Huma Qureshi, Hafsa Naeem, Sumbal Shahzadi, Zobia Sehar, Rubeena Hassan

**Affiliations:** 1 Department of Botany, The Islamia University of Bahawalpur (Baghdad ul Jadeed Campus), Bahawalpur 63100, Pakistan; 2 Department of Botany, University of Chakwal, Chakwal 48800, Pakistan

**Keywords:** plants and people, perceptions of plants, human-plant relations, plant cultural markers, wild medicinal plants

## Abstract

The current study aimed to explore the traditional knowledge and practices of indigenous communities in Bahawalpur and adjacent regions, Pakistan, to treat a variety of diseases with a particular focus on the usage of ethnomedicinal wild plants. The objective of the study was to collect and evaluate local and indigenous knowledge regarding the quantity, variety, use and management of resources by the people. Data were gathered using Rapid Appraisal Approach (RAA), which involved in-person interviews. The data were analyzed using descriptive statistics methods as well as common ethnobotanical analytical techniques viz. use value (UV), relative frequency of citation (RFC), informant consensus factor (ICF), fidelity level (FL), relative importance (RI), frequency index (FI), family use value (FUV), family importance value (FIV), popular therapeutic use value (POPUT), plant part value (PPV), preference ranking (PR), cultural significance index (CSI), rank order priority (ROP) and Jaccard index (JI). A total of 158 plant species from 49 families were identified. The perennial herbs made up 72% of the documented species of therapeutic plants. There were 21 different species in each of the two major plant families i.e., Fabaceae and Poaceae. The maximum ICF were calculated for gonorrhea and paralysis (1.0) and maximum POPUT was calculated for skin disorders (0.088) and cough (0.077), respectively. The maximum UV was found for *Rumex crispus* (0.57) and RI for *Leucaena leucocephala* (4.38). *Heliotropium crispum* had the highest FL value (83%) and is used to treat kidney diseases. *Alhagi maurorum* had the greatest RFC (0.009) and FI (0.93). Arecaceae (0.45) and Fabaceae (13.39%) had the greatest FUV while Poaceae and Fabaceae had the highest FIV (13.29 for each). Leaves were the most frequently used plant part (35%). *Alhagi maurorum* (4.0) and *Rumex crispus* (32.57) had the highest CSI and ROP, respectively. The maximum JI (28.31) was calculated for Cholistan desert. These results showed the local population’s reliance on herbal remedies to treat prevalent illnesses. To demonstrate the viability of species, it′s crucial to emphasize both the use and conservation of these species. Adopting innovative applications, enhancing their value, and prioritizing the protection of multipurpose wild plants in inhabited environments is vital.

## 1. Introduction

Ethnomedicinal research is the scientific examination of how indigenous populations utilize plants for medicinal purposes. They pay special attention to the traditions and beliefs of these tribal communities that have been using these plants for a long time. Approximately 80% of people in industrialized and developing states rely on natural remedies, notably chemical-free medicines for fundamental wellness programs [1]. As a result of the popularity of traditional herbal medicine, especially ‘tribal medicine’, ethnobotany was developed. The potential future production and implementation of novel, effective therapeutic compounds have been made possible by the discovery of previously undiscovered knowledge through ethnobotanical surveys [2]. Ethnomedicine is about studying how different cultural groups especially native people from specific places treat illnesses. It looks into traditional medicine; how different cultures use it and the cultural aspects of these healing practices. A region’s ethnomedicinal knowledge which contains knowledge on how humans employ plant species pharmacologically is a priceless cultural asset. According to the World Health Organization (WHO) herbal medicine is a combination of knowledge, skills, convictions, and practices that people from all cultures employ to heal illnesses [1]. In Pakistan almost 84% of people have utilized plants as medication. Medicinal plants are defined as ‘any plant species in which all or a part of it has pharmacological activity’ or ‘plant/plant part fresh or dried, as whole or ground, juices, gums, latex, essential oils and other fixed or similar components which are used pure or mixed on drug development by the World Health Organization and Pharmacopoeia, respectively [3]. Through the gathering of older people centuries-old traditional folk knowledge as well as the discovery of new plant species with significant medicinal and commercial value, there is an increasing interest today in enlightening function of ethnobotanical knowledge [4]. Out of 422,000 plant species, roughly 50,000 are used medicinally. According to the WHO 25% of medications that are prescribed and 11% of substances and compounds that are precursors to medications are manufactured from plants [5]. People who study how different cultures use plants and natural resources for their health often look at the connections between culture, nature, and health. These connections are mostly studied by two fields: ethnobiology and ethnomedicine. Ethnobiologists are researchers who focus on how indigenous groups and other communities understand and use plants and nature for their well-being. They come up with ideas and thoughts about how these cultures relate to their environment and health. In other words, they think about how different societies view and use nature to stay healthy [1]. To demonstrate the importance of local plant species such as in the development of new drugs, ethnobotanical research is essential [5]. The use and effectiveness of plant testing started as a result of a greater understanding of ethnobotany, which enhanced the production of food and medicine. Despite technology and globalization, natural medicine is still practiced by 60 to 85% of developing countries [6]. The usage of medicinal plants by humans has received a lot of attention and has implications for global health [7]. Ethnobotanical study is critical for demonstrating the importance of local plant species, such as in the development of new drugs [8,9]. The primary reason why plants are necessary for human survival is that they contain phytochemicals, which have a wide range of biological functions. One example of an exploratory survey technique used in ethnobotany is a biodiversity inventory utilized in the community [10]. According to cultural practices and traditional knowledge, people employ medicinal plants to treat ailments [11].

Plant-based therapy is efficient and has few adverse effects. Because the current allopathic medical system is either too expensive or insufficient in developing countries, the majority of people in rural regions rely on folk medicine [9,10,11,12]. The presence of species with high ethnomedicinal significance in Pakistan’s flora demonstrates that our biodiversity is very rich [1]. *Diospyros lotus*, *Viola pilosa*, and *Trillium govanianum* are some of the common medicinal herbs still utilized by the majority of Pakistan’s indigenous people [11]. Chinese, Ayurvedic, and Greek Arabic medicines are the three Eastern medical systems that are practiced in Pakistan. Many pharmaceuticals used by doctors today were first utilized by ancient societies such as the Egyptians, Babylonians, Greeks, Romans, Chinese, and people of the subcontinent [12].

Pakistan is Asia’s seventh-largest producer of medicinal herbs. More than 600 species are employed in traditional medicine, and over 75% of Pakistan’s native population use medicinal herbs as their primary or sole form of treatment [11]. Herbal medications have been used as therapies in the pharmaceutical industry since the dawn of time [8]. More than 75% of Pakistan’s population relies exclusively or primarily on medicinal plants to take care of their medical needs [5]. Traditional healthcare systems include ethnomedicine as a crucial component and people have long been used medicinal plants to cure a variety of illnesses. The unique ecosystem and rich cultural heritage of the Bahawalpur and surrounding regions, Pakistan have cultivated a plethora of ethnomedical knowledge. Many plants have been used by local cultures as remedies for common illnesses and health problems. To heal illnesses the people of Bahawalpur use several well-known ethnomedicinal plants, which are explored and documented in this study. By comprehending and preserving this ancient knowledge, we may be able to discover new sources of medication, support the preservation of regional biodiversity and advance medical procedures that are sensitive to cultural differences.

Bahawalpur and its adjacent areas in Pakistan are well recognized for their diverse ecosystems and rich biodiversity. As a result, they probably support a wide variety of medicinal flora that may have untapped potential for a range of therapeutic uses. Ethnomedicine and traditional medicine may have a long tradition in certain areas. Investigating local folklore about medicinal plants might provide insightful information about past applications and the prospective therapeutic qualities of diverse plant species. The identification of important plant species and the promotion of their sustainable usage can help conservation efforts by documenting the medicinal flora in these areas. Finding novel bioactive chemicals with pharmaceutical potential may result from research on the medicinal flora in these particular regions. The discovery of novel medicinal plant species or active chemicals can be of great interest to the scientific and medical sectors because many current medications have their roots in nature. Our current study’s objectives included (i) identify and investigate the important plants utilized as ethnomedicine in study area (ii) quantitatively analyze data using ethnobotanical techniques.

## 2. Materials and Methods

### 2.1. Study Area 

**Figure 1:**
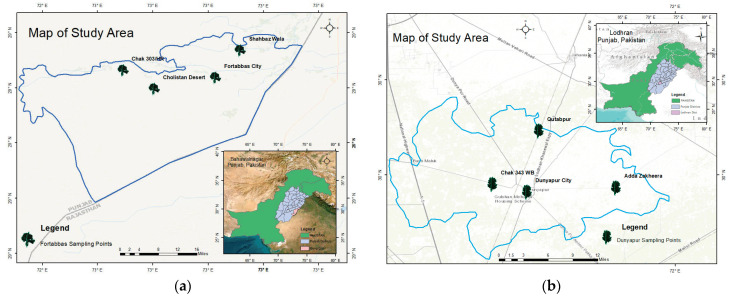

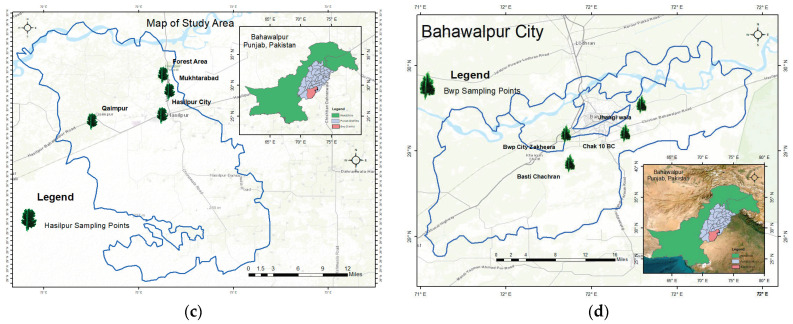
Map of study area (**a**) Fort Abbas (**b**) Dunyapur (**c**) Hasilpur (**d**) Bahawalpur.

The sampling sites of study area comprised of Bahawalpur, Hasilpur, Fort Abbas, and Dunyapur (Figure 1a–d). Bahawalpur lies in the southeast corner of Punjab Province. It is the entry point to the adjacent Lal Suhanra National Park and is situated on the edge of the Cholistan Desert. A hot desert environment, with exceptionally high temperatures and little precipitation, prevails in South Punjab, Pakistan. Second place, Hasilpur is sited on the northern boundary of the Cholistan desert. Fort Abbas is situated south of Haroonabad, near Faqirwali, on the border of Pakistan and India. Dunyapur is located on the northern side of River Sutluj and on the north of it is old bed of river Suck Bias.

### 2.2. Design of Sampling and Informant Selection

**Figure 2:**
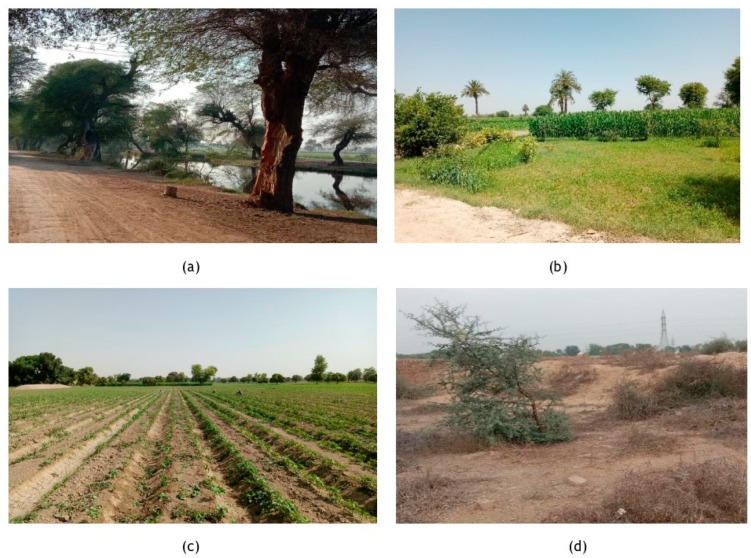
Sceneries from the study area (**a**) Fort Abbas (**b**) Duniyapur (**c**) Hasilpur (**d**) Bahawalpur.

Surveys to collect ethnobotanical knowledge were carried out in various localities from October 2022 to April 2023. We conducted interviews and made visual observations using the snowball method. We also made several field visits to meet with indigenous communities and traditional local healers known as Vaidyas (Figure 2a–d). Overall, 1813 respondents (1197 men and 616 women) were randomly selected from the study area. The questionnaire contained information on the regional names, functions, customs, ailments they treat, and strategies for herbal synthesis of medicinal flora [5]. To conduct the ethnobotanical survey the interviewees were asked questions regarding the form to use, dosage, ingredients utilized and herbal remedies for treating various diseases.

### 2.3. Data Collection and Identification of Voucher Specimen

Plant specimens were collected from the study area and, after drying in the shade, pressing, and arranging them on herbarium sheets, they were deposited in the department of Botany at The Islamia University of Bahawalpur after assigning voucher numbers. The international plant name index was used to verify the scientific names [13].

### 2.4. Methods of Quantitative Ethnomedicinal Data Analysis

The data were analyzed using standard quantitative indices. Microsoft Excel software v. 2019 and SPSS v. 25 were used for analysis of ethnobotanical data [13]. Data analysis techniques were carried out through information cross-referencing and verification.

#### 2.4.1. Informant Consensus Factor (ICF)

ICF was calculated for informants’ agreement on the reported treatment based on each category of disease. The following formula was used to calculate it [14]:

$$\mathrm{ICF}=\frac{Nur-Nt}{Nur-1}$$

where ‘Nur’ is total use reports for each category; ‘Nt’ is total number of species used for that category. 

Using the ICF, researchers can determine which use categories or traditional knowledge are more culturally significant and which are more variable or less frequently recognized within the community. This knowledge can be useful in understanding the cultural relevance of traditional practices prioritizing conservation efforts, and devising sustainable natural resource management plans. The ICF scale is 0 to 1. A number close to 1 implies a high level of agreement or consensus among informants regarding the relevance of a given use category and the plants associated with it. A score closer to 0 shows a lack of consensus, implying that informants may have various or varying ideas regarding the relevance of a certain use category or the plants used for that purpose.

#### 2.4.2. Popular Therapeutic Use Value (POPUT)

POPUT measures significance of a plant species for medicinal and therapeutic uses. The following formula was used to calculate the popular therapeutic use value [2]:

$$POPUT=\frac{NURIT}{TUR}$$

where ‘NURIT’ is number of use reports for each illness or therapeutic effect, and ‘TUR’ is total number of use reports

#### 2.4.3. Use Value (UV) 

The UV of plant species relates to their practical usefulness to human communities. The use value of a plant species varies according to its cultural, geographical, and biological context. Use value was calculated using the following formula [2]:

$$\mathrm{UV}=\sum \;\frac{Ui}{N}$$

where ‘Ui’ is number of uses recorded for a given species by each informant, and ‘N’ is total number of informants.

#### 2.4.4. Fidelity Index (FI)

The ability of a plant species to exist consistently in a particular environment is referred to as species fidelity. A higher fidelity score suggests a better relationship between a plant species and a certain habitat. A low fidelity index on the other hand, indicates that a species has a more general dispersal and occurs in a variety of habitats. The following formula was used to calculate the species fidelity index [14]:

$$\mathrm{FL}\;(\%)=\frac{Np}{N}\times 100$$

where ‘Np’ is number of species that are present in a specific category. ‘N’ is accurate sum of consumption for a particular species.

#### 2.4.5. Relative Importance (RI)

RI aids in the prioritization of plants based on their importance and prevalence in local knowledge systems. It is a useful tool for identifying significant plants with cultural, medical, or ritual significance to a society. The range of RIIs is 0 to 1, with higher values indicating more relative importance within the researched community. These indices aid in understanding the cultural significance and knowledge associated with various plants, as well as in guiding conservation efforts and supporting sustainable practices that are consistent with local customs and traditions. It was determined as [14]:

$$\mathrm{RI}=\frac{(R\cdot Ph+R\cdot BS)\;}{2}\times 100$$

where ‘R·Ph’ stands for relative pharmacological properties. ‘R·Ph’ is calculated by dividing number of uses (U) by total number of use reports. ‘R·BS’ is calculated by dividing number of diseases treated by a plant species by total number of diseases.

#### 2.4.6. Cultural Importance Index (CII)

CII aims to quantify importance of plant species based on their cultural significance considering traditional knowledge, customs, and practices associated with each plant as well as their relevance in the daily lives of the people in a particular community. The index typically ranges from 0 to 1, with 0 indicating a plant with little to no cultural significance and 1 indicating a plant with the greatest cultural significance within a community. The following formula was used to calculate the index [14]:

CII = ∑ (Ui × Fi)

where Ui is total number of different uses for a plant, Fi is cultural significance factor for the plant species.

#### 2.4.7. Frequency Index (FI)

FI is a quantitative metric used to quantify the relative importance of a specific plant species in a given community. It is a useful tool for comprehending the relevance of diverse plants in traditional knowledge systems as well as the local significance of their applications. The outcome is typically represented as a percentage. A higher FI score suggests that a plant species or its use is more important and well-known among the population polled. A lower FI, on the other hand, indicates that the plant species is less common or important in that particular cultural setting. FI was calculated as [1]:

FI = (ΣU/N) × 100

where ΣU is sum of number of different uses attributed to the plant species by different informants; N is total number of informants interviewed.

#### 2.4.8. Family Use Value (FUV)

FUV is a tool for comprehending the cultural value and significance of various plant groups in traditional knowledge systems. It assists in identifying major plant families that play important roles in the lives of distinct societies as well as possible conservation objectives. It was calculated as [1]:

$$\mathrm{FUV}=\;\frac{\Sigma UVs}{n}$$

where ‘ΣUVs’ is sum of the use values of the species within a family, and ‘n’ is total number of species within same family.

#### 2.4.9. Plant Part Value (PPV)

PPV assists in determining which plant parts are most valued and used by a particular culture. Understanding PPV is critical for the conservation and sustainable management of plant resources because it identifies which parts are most vulnerable to overharvesting and which have the greatest commercial potential without jeopardizing the plant’s survival or the culture’s traditional practices. It was calculated as [1]:

$$\mathrm{PPV}\;(\%)=\frac{\varepsilon RU(plant\;part)}{\varepsilon RU}\times 100$$

where $\in RU\;\left(plant\;part\right)\;is\;sum$ of uses reported per part of a plant, and ∈RU is total number of uses reported for all parts of plant.

#### 2.4.10. Family Importance Value (FIV)

FIV is used to evaluate relative significance of families. It was calculated as [14]:

$$\mathrm{FIV}=\frac{FC\left(family\right)}{N}\times 100$$

where ‘FC’ is number of informers revealing family, and ‘N’ is total number of informants who participated in research.

#### 2.4.11. Rank Order Priority (ROP) 

ROP is a quantitative method used to prioritize or rank plant species based on their importance or significance within a specific cultural context. This approach helps researchers understand which plants hold the highest priority or value in the traditional knowledge and practices of a community, often in relation to their various uses. ROP was calculated as [13]:

ROP=FL×RPL

#### 2.4.12. Preference Ranking (PR)

Preference rating index was used to determine which medicinal plant was best for each type of ailment. The medicinal plant that participants thought would be the most successful in curing the reported disorders would be given the highest value, while the one that they thought would be the least successful would be given the lowest value. The scores for each species were added to determine the rank. This made it possible to identify the plant that the locals use to cure the ailments that are frequently reported. Following formula was used to calculate PR [1]:

PR = ∑ (Ri × Wi)

where Ri is rank or preference score assigned to the ith plant species; Wi is the weight or importance factor assigned to ith plant species.

#### 2.4.13. Cultural Significance Index (CSI)

CSI was used to determine how well-aligned informant knowledge was with use of reports for a particular species. Following formula was used to calculate it [15]:

CSI=∑(i×e×c)×CF

where ‘i’ is management of species that have a significant impact on the community (a species that is cultivated, managed, or operated in any way receives a score of 2 and a value of 1 if the species is still free of any kind of manipulation), ‘e’ is use preference of informant for one plant species over another for specific purpose (value of 2 is for preferred species and 1 is for non-preferred species), ‘c’ is use frequency of a plant species.

#### 2.4.14. Comparison of Ethnomedicinal Data by Jaccard Index (JI)

The comparison of ethnomedicinal data with the published literature was determined by Jaccard index. This index is used to compare study data to other ethnobotanical studies undertaken in different regions around the world as well as among indigenous groups in the examined locations. The formula for calculating JI is as follows [1]:

$$\mathrm{JI}=\frac{c\times 100}{(a+b)-c}$$

where ‘a’ is recorded number of species of study area ‘A’, ‘b’ is the documented number of species of area ‘B’, and ‘c’ is common number of species in both areas.

## 3. Results

### 3.1. Socio-Demographic Features of Informants

After visiting the study sites, a total of 1813 people—1197 men and 616 women—were interviewed. In total, 158 plant species from 49 families were listed with their medicinal applications. Herbalists (301 respondents), farmers (499 respondents), Pansars (244 respondents) and others (665 respondents) were among the respondents. Among the 251 male and 169 female respondents there were 247 respondents between the age of 30–40, 624 between the age of 40–50, 609 between the age of 50–60, and 313 respondents beyond the age of 60. Illiterate respondents (21%), primary-level respondents (20%), middle-level (16%), matric-level (16%) and above-matric-level (21%) respondents were included in current study. Herbalists (17%), pansars (14%), farmers (27%) and others (36%) were among several jobs mentioned by respondents (Table 1). 

### 3.2. Enumeration of Wild Medicinal Plants 

**Figure 3:**
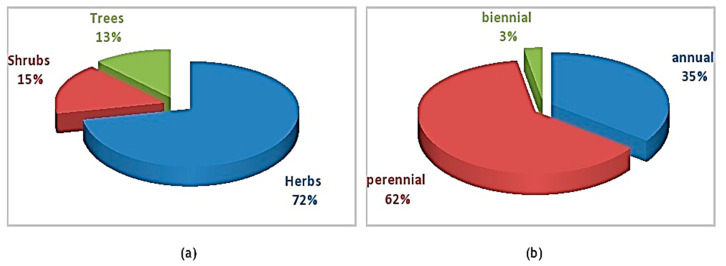
Growth habits of wild edible plants: (**a**) life form of plant species, (**b**) life span of plant species.

To count the number of plant species from each family in study area, medicinal plant enumeration was carried out. The enumeration revealed details about reported plant species’ life span and preferred habitat. Herbs made up majority of the plants (72%) and shrubs (15%) were the second-most common type of habit. Trees make up 13% of all other plants. Perennial herbs, shrubs, grasses and trees made up majority of species (62%) based on the average lifespan of plants reported from field followed by annuals (35%) and biennials (3%) (Figure 3a,b). 

### 3.3. Frequency of Plant Parts used and Methods of Preparation of Ethnomedicinal Remedies 

**Figure 4:**
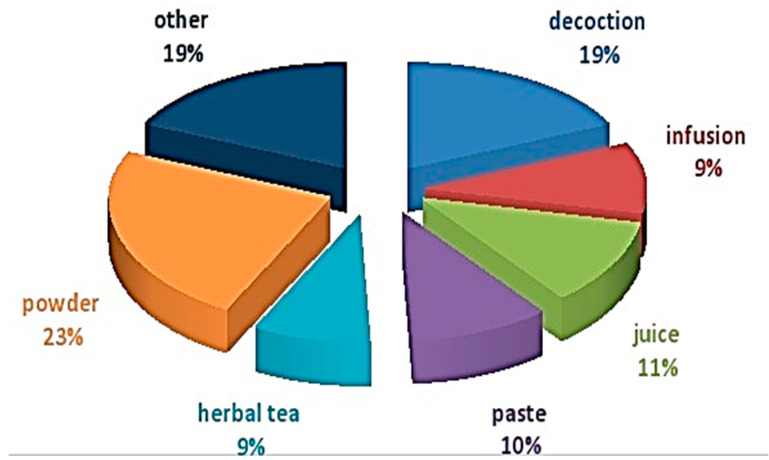
Preparation method to cure diseases.

There are several different methods used to prepare the medications. To make herbal formulations, a variety of methods have been used including use of powder (23%), decoction (19%), juice (11%), paste (10%), infusion (9%), herbal tea (9%) and others (19%) (Figure 4). Raw materials, liquids, ashes, and several other things are examples of others. 

### 3.4. Ethnomedicinal Plant Traits Used by Indigenous People 

Research in the area has revealed the traditional use of wild plants to remedy a wide range of health issues. Certain plant species are employed for treatment of diverse ailments. Using collective responses, percentage of usage for each specific disorder was calculated as shown in Table 2. 

### 3.5. Use Value (UV)

UV reveals relative significance of a plant species. The species in study area have use values ranging from 0.16 to 0.57. *Rumex crispus* had highest use value (0.57), while *Dalbergia rostrata* had lowest value (0.15) (Table 2). Just because a plant has lower UV does not make it any less significant; rather, it may be that traditional healers have not used it as much. A plant with a high UV implies that there are more reports about it, and a plant with a low UV reveals that there are fewer reports about it from the respondents. The plant’s maximal use value reveals how well-known it is among the community and how well it may be used to treat illnesses. A high UV indicates that the plant is utilized more locally; low UV indicates that plant is used less frequently in that location. *R. crispus* has been used to treat kidney stones, skin disorders, wound healing and urinary disorders. 

### 3.6. Fidelity Level (FL)

FL is used to identify species that locals prefer to utilize to treat certain illness in comparison to other species that are also being used to treat same illness (Table 2). In study area, 158 species have been reported that are utilized to cure various illnesses. The values for FL ranged from 23% to 83%. *Heliotropium crispum* had highest FL value (83%), followed by *Malcolmia africana* and *Malva sylvestris* (each 80%). *Senna occidentalis* FL value was found to be the lowest (23%). 

### 3.7. Relative Importance (RI)

Highly adaptable species with high RI values is utilized to treat a variety of illnesses. These plants have high RI values because locals are quite knowledgeable about them and use them to heal various illnesses. *Leucaena leucocephala* (4.38) has highest RI value. This plant species in current study is reported to treat cancer, fever, jaundice, constipation and diabetes. However, species with lower RI values, such as *Spergula arvensis* (1.78), *Aeluropus lagopoides* (1.8) and *Cenchrus ciliaris* (1.8) are probably less significant with less medicinal value in local community (Table 2). It was demonstrated that species with high RI values were frequently employed to treat a wide range of illnesses. 

### 3.8. Relative Frequency Citation (RFC)

In our work, RFC ranges from 0.003 to 0.009. The highest RFC (0.009) of *Alhagi maurorum* suggests that this species is frequently used by locals to cure a range of medical conditions. Due to prominence of this plant in study area, people are quite familiar with it. The plants with lowest RFC values are *Gamochaeta pensylvanica*, *Verbascum virgatum, Rumex crispus, Cenchrus ciliaris* and *Spergula arvensis* (0.003 for each). RFC value which depicts the relative importance of plant species is influenced by percentage of informants who report utilizing a species (Table 2).

### 3.9. Frequency Index (FI)

FI is a metric for determining the relative significance of various plant species in a cultural setting (Table 2). The more frequently a plant species is noticed and considered to be abundant within study area, higher the FI value. Its value ranges between 0.38 and 0.93. *Alhagi maurorum* was found to have highest value (0.93). *Gamochaeta pensylvanica*, *Rumex crispus*, *Spergula arvensis*, *Centaurium pulchellum* and *Verbascum virgatum* had lowest values (0.38 for each). 

### 3.10. Rank Order Priority (ROP)

Rank order priority is used to order species in a list based on how well-known they are for treating various ailments. From 32.57 (highest priority) to 0.13 (lowest priority), rank of priority was determined. *Rumex crispus* had highest value (32.57), showing that it was most effective in area for treating particular illnesses. Other plants with high ROP values included *Verbascum virgatum* (24.43), *Eragrostis cilianensis* (21) *Emex spinosa* (20.29), *Spergula arvensis* (20.29), and *Polypogon monspeliensis* (20.0) while *Carthamus oxyacantha* possessed the lowest ROP i.e., 0.13 (Table 2). 

### 3.11. Plant Part Value (PPV) 

**Figure 5:**
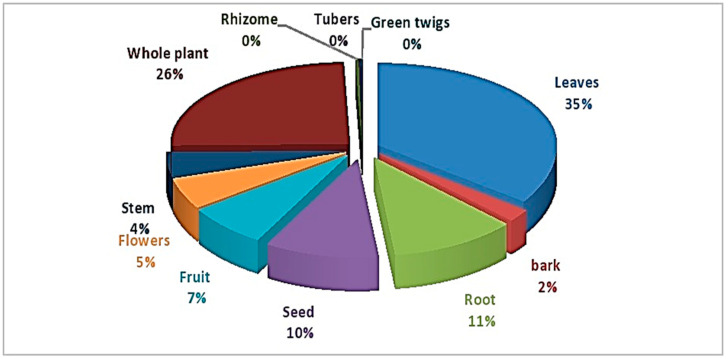
Plant parts used in herbal remedies.

People use different plant parts (alone or in combination) to treat a variety of illnesses. In total, eleven different plant parts were used. The majority of plant parts were found to be leaves (35%), the whole plant (26%), roots (11%), seeds (10%), fruit (7%), flowers (5%) and stems (4%) (Figure 5).

### 3.12. Cultural Significance Index (CSI) 

CSI is determined to reflect the cultural significance and worth of different plant species to human communities (Table 2). Cultural significance of each species varies amongst different communities. CSI score ranged from 0.467 to 4, with higher values denoting a plant’s greater cultural relevance. *Alhagi maurorum*, *Poa annua, Lysimachia arvensis, Cuscuta reflexa* and *Gamochaeta pensylvanica* were found to have highest values (4 for each). *Cenchrus biflorus* and *Spergula arvensis* were found to have lowest values (0.466 for each). 

### 3.13. Informant Consensus Factor (ICF)

ICF is based on the responses given by the locals of the study region, measures extent of relationship between diseases and particular plant species that are being utilized to treat particular ailments (Table 3). Locals from study area have reported using 158 plant species from 49 plant families. ICF varied from 0.26 to 1.0, indicating that there is strong agreement among informants about use of plants as medicine. The highest ICF (1.0) was recorded for headache, heart disease, hepatitis, blood pressure, back pain, earache, appetizers, epilepsy, antimicrobial gargles, gonorrhea, hair treatment, hemorrhage, insomnia, muscle cramps, osteoporosis, paralysis, snake-bite, anti-tumor, typhoid and worm infestation indicating that fewer plants are used to treat these ailments. For skin disorders, minimum ICF (0.26) was recorded. 

### 3.14. Popular Therapeutic Use Value (POPUT)

To measure significance of a plant species for therapeutic and medicinal uses, popular therapeutic use value is calculated. Locals in our study area responded and POPUT ranged from 0.001 to 0.088. According to Table 3, skin disorders had highest reported POPUT (0.088) followed by cough (0.077), fever (0.066), asthma (0.06) and constipation (0.052). This implies that community relies extensively on medicinal plants to treat conditions indicated above. For gargles and headaches lowest POPUT was reported (0.001). Out of 158 species in study area, 41 species were used to treat skin disorders. 

### 3.15. Family Use Value (FUV)

To determine the significance of plant families, the family usage value is determined. FUV varies in the study area from 0.18 to 0.45 (Table 4). Family Arecaceae had highest FUV followed by Asclepiadaceae (0.44), Scrophulariaceae (0.43), Resedaceae (0.38) and Gentianaceae (0.37). Ranunculaceae and Malvaceae (0.20 for each) had lowest FUVs. The community uses 21 plant species from Poaceae and Fabaceae to treat a range of ailments. Asteraceae, with 14 was family with second greatest number of species followed by Amaranthaceae, Solanaceae and Polygonaceae (9 species for each). Ranunculaceae and Malvaceae recorded lowest FUVs (0.2 for each).

### 3.16. Family Importance Value (FIV)

FIV varies in the study area from 0.63 to 13.29%. With 21 species, Fabaceae family had the highest FIV (13.29%), followed by Poaceae (13.29%), Asteraceae (8.86), Amaranthaceae, Solanaceae and Polygonaceae (5.69% for each). Ranunculaceae, Papaveraceae and Tamaricaceae (0.63% for each) had lowest FIVs. 

### 3.17. Preference Ranking (PR)

PR determined which medicinal plant was best for certain ailment. Participants were asked to rate which medicinal plant they thought was most or least efficient at curing listed ailments (Table 5). They were then asked to rate each plant according to how well they thought it would treat diseases. According to scores of research area for skin disorders *Melia azedarach* (25.0) and *Peganum harmala* (24.0) were two plants used frequently. 

### 3.18. Comparison of Ethnomedicinal Data by Jaccard Index (JI)

After comparing the ethnomedicinal data collected from the study area with the other 30 studies, it was found that the percentage of species with common uses ranges from (0%) to (28.31%). The collected data were compared with 30 previously published studies from the year 2012 to recently published data in 2023 (Table 6).

## 4. Discussion

The current study was conducted in Bahawalpur, a district located in Pakistan′s Southern Punjab province and also considered the surrounding regions of Fort Abbas, Hafizabad and Dunyapur. Random interviews were conducted. In previous study, the two plant parts that were employed the most commonly were seeds (16%) and leaves (65 reports, 28.88%) [1,8]. While it was found in an ethnobotanical study that many traditional recipes are made either with or without a supplementary substance depending on the life- form and plant part used in crude preparation for respiratory disease including decoction (71.4%), extract (66.7%), infusion & paste (38.1%), powder (33.3%) and juice & ash (14.3%) [27]. The leaves were used in 38% of traditional medical treatments. The roots (10%), stems (8%), fruits, seeds and flowers (7% each), bark (5%), branches & rhizome (2% each) were second most common plant parts, followed by whole plant (14%) and the stem (10%) [13].

Herbs were used most frequent accounting for 27 (51%) species in a survey to examine phyto-cultural diversity related to ethnomedicinal uses in Shigar Valley (Central Karakorum Ranges), Northern Pakistan [28]. This was followed by shrubs (21.40%), trees (4.7%), and sub-shrubs (2.85%). Sixty-two percent of species documented in an ethnobotanical survey in Thana village, Malakand District were herbs. Trees comprised 25% of all reported species whereas shrubs contributed 13% of the total [29]. Perennial plants made up 72% of listed plant species, whereas annual plants made up 28% according to an ethnobotanical study [5]. The most frequent types of growth were perennial plants (43%), herbs (23%), shrubs (16%) and trees (15%). Herbs made up majority of vegetation in studies from Cholistan, contributing 61.30%, followed by shrubs (18.30%), trees (8.60%), grasses (7.50%), sedges (2.20%), and climbers (2.20%).

Plants may have high UVs because they are widely available and recognized by local healers making them greatest treatment alternative [2]. In Lakki Marwat District, highest reported use value was 0.98, while the lowest was 0.10 [7]. In Vehari District, UV was estimated for plants *Citrullus colocynthis* (0.27), *Chenopodium album* (0.50), *Calotropis procera* (0.52) and *Chenopodium murale* (0.69) [8]. *Conyza canadensis* (0.58) has the highest UV [22]. Due to informants’ lack of familiarity with certain plant species and their limited understanding of their ethnobotanical applications, low UVs were found for some plants. *Alhagi maurorum, Eclipta prostrata* and *Trianthema potulacastrum* had UVs as low as 0.01 and as high as 0.22 for *Azadirachta indica* [22]. 

Plants with higher FL values are frequently utilized to treat a particular ailment [14]. *Heliotropium crispum* was used to treat a variety of illnesses including ulcers and kidney problems. The two plants with the highest FL values for treating diabetes are *Caralluma tuberculate* (61.22%) and *Artemisia scoparia* (55.56%), according to an ethnobotanical survey conducted in Kurram Agency, Pakistan [30]. *Aloe vera* had lowest FLI (46.6%) and *Brassica campestris* had highest (83%) FL value. *Brassica campestris* is a plant native to Azad Jammu and Kashmir and is used to treat skin disorders and hyperglycemia. *Azadirecta indica* has the highest value for blood purification (93.4%). *Ziziphus mauritiana* FL percentage for diabetes in Jammu & Kashmir is 98% [31]. The FL values of 129-plant species from Hafizabad District ranged from 14.3 to 100% [13].

A plant species RI value determines how many diseases and disorders it can treat [14]. *Garcinia aristata* had lowest RI value in Lahore District, whereas *Tabernaemontana divaricata* had highest [32]. *Thymus linearis* (1.06), *Caralluma tuberculata* (1.20), *Cassia fistula* (1.10), *Artemisia absinthium* (1.34), *Withania coagulans* (1.63) and other plants were the plants in the Kurram Agency with highest relative importance [11]. This finding opens the door to new uses for the plants. *Juglans regia* (0.95), *Tilia platyphyllos* (0.93), *Rosa canina* (0.84) and *Thymus* sp. (0.84) are species with high RFC values. *Mentha piperita* is utilized extensively both in Turkey and throughout the rest of the world due to its therapeutic benefits and pleasant aroma. In addition to being widely used as a spice, it is frequently utilized in Kahramanmaras to prevent colds, stomachaches and bad breath [33].

The high RFC of *Viola canescens* (0.97) in AJK suggests that locals frequently use this plant to cure a range of medical disorders [1]. *Rosa indica* had greatest RFC in Lahore District while *Deutzia scabra* and *Euonymus japonicus* had lowest values [32]. RFC value for *Centaurium pulchelum* in our study was greatest (0.037) whereas RFC values for *Gisekia pharnaceoides*, *Lasiurus scindicus* and *Alternanthera caracasana* were lower. The RFC which depicts relative importance of plant species is influenced by number of informants who report utilizing a species [33]. RFC ranged from 3 to 17% for reported species. *Triticum aestivum* (0.15), *Solanum surattense* (0.17) and *Eclipta alba* (0.15) had the highest RFCs among other plants [13].

The frequency index is a quantitative metric used to quantify the relative importance of a specific plant species in a given community [1]. *Elephantorrhiza elephantina* had the highest frequency index value reported in South Africa (35), and *Aloe grandidentata* (2) had the lowest value [34]. The frequency index of medicinal plants was calculated during an ethnobotanical survey in Nepal to evaluate how frequently people use them for ethnobotanical purposes. *Ricinus communis* had the highest value (86.41) and *Citrus limon*, *Camellia sinensis*, *Moringa oleifera*, *Artocarpus lakoocha* and *Dolichos lablab* had the lowest values (1.22 for each) [14]. *Achyranthes aspers* has a frequency index of 14.81, *Centella asiatica* (30.86), *Dioscorea bulbifera* (74.0), *Mimosa pudica* (13.58) and *Jatropha curcas* (7.40) are some of the other species used in this region [33].

Rank order priority (ROP) was computed for plant species in Bhimber District, Azad Jammu and Kashmir [31]. The ROP had values between 0.13 and 18. *Convolvulus arvensis* (0.13) had the lowest ROP value, while *Ranunculus laetus* (18.0) had the highest ROP value. Hafizabad District reported that ROP, a quantitative study of medicinal plants, was determined in an ethnobotanical survey. The values of the various plant species revealed their medicinal significance and how well-liked they are among the locals [13]. Many species’ ROP levels were greater than 75.0. The plant species with various FL values are properly ranked using the ROP index. Only eight species reached ROP values above 50 according to the subsequent RPL values reported, which were utilized as a correction factor to adjust the FL values. *Ranunculus laetus* (18.0) had the greatest ROP value and *Convolvulus arvensis* (0.13) had the lowest ROP value [32].

Studies from Bhimber, Azad Jammu, and Kashmir claim that different plant components were used to make herbal cocktails [9]. The most often employed plant parts in herbal remedies were leaves, followed by whole plant and the roots (30.2, 16.6 & 14.4%, respectively). The people preferred leaves because they are convenient to gather and frequently available. The most utilized part in terms of proportion, was the leaf (61.5%), followed by the fruit, stem, and rhizome (10.3% each), gum and bulb (7.4%) [35]. The root, leaves, bark, and fruit were the most frequently used plant components, followed by the entire plant, according to studies from Cholistan. In the research area, both as a whole and in specific regions, plants are frequently used to cure a variety of ailments [1]. Thirty-five different entire plant species are utilized to treat a variety of diseases in the Cholistan desert. A variety of medications were made from leaves of 17 different plants. The residents of the research area prepare remedies from 15 different plant parts for a range of illnesses. The most often used plant part among them similar to our findings (Table 2), was leaves.

The value of cultural importance index (CSI), which ranged from 0.13 to 8.00, was significantly influenced by the preferences, management strategies, and usage patterns of the local population [15]. The difference was influenced by the level of knowledge, cultural backgrounds, and specific local situations. Six species had CSI scores of *Oryza sativa* (0.81), *Poa annua* (7.69), *Cynodon dactylon* (7.34), *Avena sativa* (7.33), *Saccharum officinarum* (7.14), and *Zea mays* (6.77). The grass species with the lowest CSI values were *Avena sterilis* (0.15), *Lolium temulentum* (0.13), *Polygonum punctatum* (0.15), *Paspalum dilatatum* (0.14), *Pennisetum typhoideum* (0.15), and *Schismus arabicus* (0). The effectiveness and breadth of a species’ use in the treatment of a disease determines the CI value [1]. *Lawsonia inermis* (Mardan), *Piper nigrum* (Swabi), *Plantago major* (Peshawar), and *Senna alexandrina* had the highest documented cultural values for a single species in a single district, each at 1.05. 

In Hafizabad District, the informant consensus factor (ICF) value for skin disorders was reported to be 0.39 [13] but in our study, it was calculated to be 0.26. The most-often treated issues were stomach ulcers, bowel disorders, urinary problems, and skin issues, with an ICF of 1 followed by leucorrhea (0.89) and vomiting (0.86) [5]. The highest ICF was for wound healing (0.87), followed by skincare (0.85). A value of 0.73 was determined for wounds, which is essentially the same as other research but slightly less than the previously reported value of 0.87 [14]. The high value of ICF in our study indicated that participants generally agreed that fewer plant species were employed to treat particular illnesses. Our computed value (0.68) for diabetes is marginally higher than the reported value (0.53) from Toba Tak Singh [36].

POPUTs were determined for shortness of breath (0.11), abdominal discomfort (0.10), wounds (0.08), and stomach disorders (0.07). For diabetes, the POPUT was 0.04 [13], whereas in the current study it is 0.03. The POPUT was 0.14 for digestive disorders, 0.06 for colds, 0.05 for cough, and 0.04 for eczema and rheumatism [2]. According to our research, the value for cough is 0.07, which is among the highest value.

The FUV of families has been computed in Indonesia using the total number of species within that family [35]. The high FUVs may be due to the research area’s extensive use of the plant species [1]. Northeastern Morocco was the site of an ethnobotanical investigation. The distribution of the botanical families of medicinal species in the study area had a significance value ranging from 0.0023 to 0.161 [37]. Regarding the family use value of the plants recorded in this paper, the results show a high score for Caryophyllaceae (0.163), followed by Lamiaceae (0.106), Apiaceae (0.099), Rhamnaceae (0.084), Asteraceae (0.083), Poaceae (0.074), Asteraceae (0.071), Zingiberacee (0.060), Rutaceae (0.044), Thymelaeaceae, and Urticaceae (0.036 for each), Cucurbitaceae (0.034), and Ericaceae (0.024). The use values for the other families are less than 0.024. The natives employ 27 plant species and 21 genera from the Lamiaceae, Asteraceae, Cupressaceae, and Ephedraceae plant groups to treat a range of diseases [38]. The Lamiaceae and Asteraceae families each of which include 12 species, were followed by the Cupressaceae (2 species) and Ephedraceae (1 species) family.

The uses of medicinal plant species from the Asteraceae and Poaceae families were noted, which is comparable to ethnomedicinal flora described from other regions of Pakistan and the rest of the world. A total of 86 plant species, representing 38 families, have been identified in Pothwar and Cholistan, respectively [39]. The natural flora of Haroon Abad, the district of Bahawalnagar, has 81 species in 21 families [18]. With 15 species, the Poaceae family is the largest. The next largest families were Amaranthaceae (7 species), Asteraceae (7 species) and Euphorbiaceae (8 species). In the Toba Tek Singh District, weeds have evolved into 16 different plant families. The quantity of weeds in each family determined the order of the families. Asteraceae came in second with four species followed by Amaranthaceae and Poaceae with five each, Euphorbiaceae with two, and all other families found in the area with just one weed species [36]. 

Based on research conducted in Pakistan and worldwide, it was found that people in different places used plants from the Fabaceae and Poaceae families for medicinal purposes in a similar way [13]. Interestingly, 21% of the medicinal plants found in the study region were utilized to treat stomach discomfort. On a scale of one to five, eight key informants were asked to rate the potency of each of these medicinal herbs [1]. The most popular remedy for stomach issues was *Rumex nepalensis* followed by *Ruta chalepensis*, *Clausena anisata*, *Nicotiana tabacum* and *Zingiber officinale*. The strongest medicinal plant, *Allium sativum* came out on top after a preference assessment of six plants that can treat malaria [40].

The highest value of the Jaccard index (28.31) was calculated for Cholistan desert and the lowest value (0.0) was calculated for China. A lower value indicates less similarity between the data of two areas and a high value indicates a high similarity between the two areas. It may depend upon the presence of common species or common uses of the species from both study areas [35]. In this study, the respondents have reported the unique species or unique uses of the species. The maximum number of common species recorded in Cholistan desert was 62.0 [1]. The lowest percentage (0%) of species with common uses was calculated for China [26]. The maximum percentage of species with common uses was recorded in Yazman [17]. The differences and similarities in ethnomedicinal research demonstrate the importance of traditional knowledge in many contexts where historical, organoleptic and phytochemical elements interact to influence their choice.

## 5. Conclusions

In conclusion, our exploration of the wild medicinal plants used for basic healthcare by the local people of Bahawalpur and its neighboring regions in Pakistan has provided valuable insights into the traditional knowledge and practices of these communities. Through our study, we identified a diverse range of plant species that play a crucial role in addressing basic health needs. These findings underscore the importance of preserving and promoting the sustainable use of indigenous flora in the context of healthcare. As we observed, traditional knowledge is at risk of being lost among the younger generation, emphasizing the need for conservation efforts and cultural transmission. Additionally, our research highlights the potential for further investigations into the biological activities and bioactive components of specific plant species, which could contribute to the development of effective and culturally relevant healthcare solutions. In summary, this study serves as a foundation for the protection and sustainable utilization of wild edible plants in Bahawalpur and adjacent regions, contributing to both the preservation of traditional knowledge and the improvement of community health practices.

## Figures and Tables

**Table 1:** Socio-demographic data about informants of the study area.

Variables	Demographic Categories	Numbers	Percentage
Gender	Male	1196	66%
	Female	617	34%
Age	31–40	247	13%
	41–50	624	34%
	51–60	609	34%
	>60	313	17%
Education	Illiterate	383	21%
	Primary	378	20%
	Middle	286	16%
	Matric	393	21%
	Above matric	373	21%
Occupation	Herbalist	301	17%
	Farmer	499	27%
	Pansar	244	14%
	Others	665	36%
	Housewife	104	6%

**Table 2:** Ethnomedicinal plants used by indigenous people of study area.

BN	LN	Family	LF	PU	Rec	App.	Uses	UV	FL	ROP	CSI	RFC	FI	RI
*Abutilon indicum* (L.) Sweet	Kanghi booti	Malvaceae	PS	Seeds, flowers, leaves, whole plant	Paste, infusion	Massage, oral	Piles, anti-inflammatory, cancer, diarrhea	0.28	31	18.75	3.5	0.007	0.77	3.56
*Acacia ampliceps* Maslin	Australian kikar	Fabaceae	PT	Whole plant, leaves	Herbal tea, powder, juice	Oral	Skin disorders, digestive disorders, diabetes	0.37	50	16.67	1.06	0.004	0.44	2.6
*Achyranthes aspera* L.	Puthkanda	Amaranthaceae	PH	Whole plant	Decoction, herbal tea, powder	Oral	Asthma, fever, lung infections, gastrointestinal diseases	0.25	38	10.5	3.76	0.008	0.88	3.42
*Adiantum capillus-veneris* L.	Khoo_boti	Pteridaceae	PH	Whole plant	Decoction, paste, powder	Oral, topical	Lung infections, animal bites, flue	0.25	42	10.5	2.58	0.006	0.66	2.65
*Aeluropus lagopoides* (L.) Trin. ex Thwaites	Pooji Chabbar	Poaceae	PG	Whole plant, stem, fruit	Herbal tea, paste	Oral, topical	Diabetes, wound healing	0.25	62	10.61	0.5	0.004	0.44	1.8
*Aerva javanica* Juss.	Booh	Amaranthaceae	PS	Whole plant, leaves, seeds	herbal tea, powder, paste	Oral, massage	Skin disorders, arthritis, kidney stones	0.23	46	11	3.47	0.007	0.71	2.73
*Albizia lebbeck* (L.) Benth.	Shirin	Fabaceae	PT	Seeds, leaves, flowers	Seeds, powder, tea, decoctions	Massage, topical	Eye diseases, skin disorders, wound healing, pain killer	0.33	33	7.23	3.2	0.006	0.66	3.5
*Alhagi maurorum* Medik.	Jawasa	Fabaceae	PS	Whole plant	Powder, herbal tea, decoctions	Oral	Fever, diarrhea, vomiting	0.17	41	16	4	0.009	0.93	2.6
*Alternanthera caracasana* Kunth	Khaki_weed	Amaranthaceae	PH	Whole plant	Decoction	Oral, topical	Headaches, vomiting, skin diseases, animal bites	0.4	40	16	1.17	0.005	0.55	3.56
*Alternanthera philoxeroides* (Mart.) Griseb.	Alligator-weed	Amaranthaceae	PH	Leaves, flowers	Herbal tea, decoction	Oral	Asthma, diabetes, influenza, hepatitis	0.4	30	12	2.5	0.005	0.55	3.45
*Amaranthus viridis* L.	Ganar	Amaranthaceae	AH	Leaves, whole plant, and roots	Powder, herbal tea	Oral	Cough, diarrhea, diabetes, constipation	0.27	33	8.91	3.75	0.008	0.82	2.79
*Asphodelus tenuifolius* Cav.	Piazzi	Asphodelaceae	AH	Seeds, roots, stems, leaves	Juice, herbal tea, decoctions	Oral, topical	Wound healing, diuretics, piles	0.27	54	14.72	2.75	0.006	0.6	2.68
*Azadirachta indica* A. Juss.	Neem	Meliaceae	PT	Seeds, fruit leaves, seeds	Powder, infusion	Oral, topical	Blood purifier, skin diseases, toothache, gastrointestinal diseases	0.23	40	14.5	3.25	0.007	0.71	2.73
*Bauhinia variegata* L.	Kachnaar	Fabaceae	PT	Flowers, leaves, roots, fruit	Decoction, powder, herbal tea	Oral	Blood pressure, stomach disorders, throat infections, diarrhea	0.36	40	16	2.58	0.006	0.6	3.45
*Boerhavia diffusa* L.	It-sit	Nyctaginaceae	PH	Roots, leaves	Juice, powder	Oral, topical	Jaundice, eye diseases, diuretics, diabetes	0.4	40	16	2.35	0.005	0.55	3.47
*Boerhavia procumbens* Banks ex Roxb.	Pookhla	Nyctaginaceae	PH	Leaves, roots seeds, flowers	Powder, decoction, herbal tea	Oral	Anti-inflammatory, blood purifier, jaundice	0.36	36	13.08	2.75	0.006	0.6	3.47
*Calligonum polygonoides* L.	phog	Polygonaceae	PS	Green twigs and whole plant	Powder	Oral, massage	Stomach disorders, rheumatism, toothache	0.27	36	9.72	2.75	0.006	0.6	2.68
*Calotropis gigantea* (L.) Dryand.	Bara_ak	Apocynaceae	PS	Whole plant, roots, bark	Juice, fresh paste	Oral, topical, massage	Cough, asthma, fever	0.33	50	16.5	1.5	0.006	0.66	3.5
*Calotropis procera* (Aiton) Dryand.	Aak	Apocynaceae	PS	Leaves, flowers	Dried flowers, leaves, juice	Oral, massage	Arthritis, asthma, digestive disorders	0.3	50	14.15	2.5	0.005	0.55	2.68
*Capparis decidua* Edgew.	Karri deela	Capparaceae	PS	Fruits, seeds	Fresh juice, paste	Oral, topical	Production and purification of blood, arthritis, cough, asthma	0.3	30	14	3.25	0.007	0.71	3.5
*Capparis spinosa* L.	Kuber	Capparaceae	PS	Fruits, leaves, roots, bark	Herbal tea, powder, paste	Oral, topical, massage	Diabetes, cough, asthma	0.3	30	13.8	1.73	0.007	0.71	3.53
*Carthamus oxyacantha* M.Bieb.	Pohli	Asteraceae	AH	Whole plant, seeds, leaves	Juice, decoction	Oral, massage	Ulcers, jaundice, skin diseases, diarrhea	0.33	42	0.13	3.2	0.006	0.66	3.5
*Cassia fistula* L.	Amaltas	Caesalpiniaceae	PT	Leaves, fruits, seeds	Powder, decoction, herbal tea	Oral	Arthritis, skin disorders, fever, constipation	0.3	30	16.72	3.25	0.007	0.71	3.53
*Cenchrus biflorus* Roxb.	Pasra	Poaceae	AG	Whole plant, seeds	Dried seeds powder, fresh paste of whole plant	Oral	Asthma, cough, diuretic	0.22	62	15.43	0.52	0.004	0.6	3.47
*Cenchrus ciliaris* L.	Dhaman	Poaceae	PH	Whole plants, leaves	Leaf infusion, decoction	Oral	Pain killer, urinary diseases, kidney stones	0.42	67	14.25	0.466	0.003	0.44	1.8
*Cenchrus setigerus* Vahl	Kali-dhaman	Poaceae	AH	Whole plant	Paste, infusion, powder	Oral, topical	Fever, skin disease	0.36	46	16.56	2.92	0.006	0.49	2.02
*Centaurium pulchellum* (Sw.) Druce	Barik chirayata	Gentianaceae	BH	Leaves	Powder, decoction	Oral	Jaundice, fever	0.25	36	14.8	0.5	0.004	0.38	2.57
*Chenopodium album* L.	Bathu	Chenopodiaceae	AH	Seeds, leaves, shoots	Decoction, herbal tea, powder	Oral, massage	Stomach laxative, diuretic, hepatitis	0.25	57	14.25	1.6	0.006	0.66	2.71
*Chenopodium murale* L.	krund	Chenopodiaceae	AH	Leaves, seeds, stem, whole plant	Fresh paste of leaves, dried seed powder, herbal tea of stem	Oral	Digestive disorders, diarrhea, constipation, ulcers, diuretic	0.28	50	19.23	3.5	0.007	0.77	3.56
*Cirsium arvense* (L.) Scop.	leh	Asteraceae	PH	Leaves, whole plant	Powder, decoction, Paste	Oral, massage, topical	Cough, diuretic, bronchitis, wounds healing	0.38	35	13.5	3.468	0.007	0.71	4.32
*Cistanche deserticola* Ma	Geedar tobbaco	Orbanchaceae	PH	Whole plant, leaves decoction	Herbal tea, dried powder	Oral, massage	Arthritis, diarrhea, cough	0.36	38	14.25	1.5	0.006	0.6	2.68
*Citrullus colocynthis* (L.) Schrad.	Kor tumma	Cucurbitaceae	PH	Seed, fruits, roots, leaves	Powder, paste, herbal tea	Oral, topical	Blood purifier, skin disease, diabetes, hypertension	0.37	45	16.65	1.06	0.004	0.44	2.6
*Cleome brachycarpa* M.Vahl ex Triana & Planchon	Kastoori	Cleomaceae	PH	Whole plant, roots, leaves, flowers	Decoction, paste	Oral, topical, massage	Skin diseases, antimicrobial, rheumatism, anti-inflammatory	0.27	38	10.26	3.75	0.008	0.82	2.79
*Cleome pallida* Kotschy	Phel	Capparidiaceae	AS	Flowers, leaves	Powder, infusion, decoction	Oral	Blood purifier, Jaundice, asthma	0.3	46	13.84	3.25	0.007	0.71	3.53
*Convolvulus arvensis* L.	Hiran_khuri	Convolvulaceae	PH	Leaves, roots, flowers	Infusion, herbal tea, paste	Oral, massage	Skin diseases, constipation, blood purification	0.28	33	9.4	3.5	0.007	0.77	3.56
*Crotalaria burhia* Buch.-Ham. ex Benth.	Chag	Fabaceae	PS	Whole plant	Decoction, Paste, herbal tea	Oral, topical	Skin diseases, constipation, swelling, anti-inflammatory	0.2	40	8	1.334	0.005	0.55	1.86
*Cucumis melo* L.	Chibbar	Cucurbitaceae	AH	Whole plant, seeds, leaves, bark, flowers	Herbal tea, infusion, decoction, paste	Oral, topical	Cholesterol, constipation, kidney diseases, diuretic	0.36	70	7.6	1.375	0.006	0.6	2.68
*Cuscuta reflexa* Roxb.	Akas bail	Convolvulaceae	PH	Whole plant, stem	Essential oil of stems, infusion of whole plant	Oral, massage, topical	Painkiller, paralysis hair treatment	0.25	36	9	4	0.008	0.88	3.61
*Cymbopogon jwarancusa* (Jones ex Roxb.) Schult.	Khawi	Poaceae	PH	Whole plant, roots, shoots, seeds	Paste, decoction	Oral, topical, massage	Gargles, skin diseases, cough, anti-inflammatory	0.23	37	16.15	3.058	0.007	0.71	2.73
*Cynodon dactylon* (L.) Pers.	Khabal grass	Poaceae	PG	Whole plant, leaves, roots	Leaf paste, dried powder whole plant	Oral, topical	Wound healing, digestive disorders, eye diseases, skin disorders	0.28	35	9.99	3.5	0.007	0.77	3.56
*Cyperus conglomeratus* Rottb.	Deela	Cyperaceae	PH	Root, leaves, fruit	Decoction, paste	Topical, massage	Stomach, diuretic, constipation	0.28	36	10.29	3.73	0.007	0.77	3.56
*Cyperus rotundus* L.	Murak	Cyperaceae	PH	Tubers, leaves, roots, whole plant	Herbal tea and powder	Oral	Ulcers, stomach disorders, anti-inflammatory, fever	0.28	50	14	3.5	0.007	0.77	2.76
*Dactyloctenium aegyptium *(L.) Willd.	Madana grass	Poaceae	AH	Whole plant, leaves, seed	Juice, paste, decoction	Oral, topical	Digestive disorders, fever	0.27	33	8.91	3.75	0.008	0.82	3.58
*Dalbergia rostrata* Hassk.	Tali/shisham	Fabaceae	PT	Fruits, leaves	Decoction, powder	Oral, topical	Used for Sexual disorderses, asthma, blood purification, eye diseases	0.15	69	10.62	3.46	0.007	0.71	1.94
*Datura metel* L.	Datura	Solanaceae	AS	Whole plant, seed, leaves, flowers	Decoction, infusion, paste	Oral	Skin diseases, asthma, anti-inflammatory, cough	0.4	40	16	2.35	0.005	0.55	3.47
*Datura* spp.	Datura	Solanaceae	AH	Whole plant	Herbal tea, infusion, decoction	Oral, massage	Earache, cough, wound healing	0.3	46	13.8	3.25	0.007	0.71	3.53
*Desmostachya bipinnata* (L.) Stapf	Dab	Poaceae	PG	Whole plant, roots	Juice, paste	Oral,	Anti-inflammatory, fever, asthma	0.21	43	9.21	1.866	0.007	0.77	2.76
*Dichanthium annulatum* Stapf	Khew/khurd	Poaceae	AH	Leaves, stem	Infusion, powder	Oral	Diarrhea and Sexual disorderses	0.27	63	17.18	2.75	0.006	0.6	2.68
*Digera muricata* Mart.	Taandla	Amaranthaceae	AH	Whole plant	Paste, decoction, herbal tea, juice	Oral, topical	Constipation, diuretic, liver, diabetes	0.28	43	12.29	3.73	0.007	0.77	3.56
*Digera muricata* subsp. *trinervis* C.C.Towns.	Taandla	Amaranthaceae	AH	Whole plant, leaves, seeds, flowers	Paste, juice, infusion	Oral, topical	Wound healing, female disorders, skin disorders, arthritis	0.18	64	11.6	2.58	0.006	0.6	1.86
*Dodonaea viscosa* Jacq.	Aliar	Sapindaceae	PS	Bark, leaves, and seeds	Powder, paste, decoction	Oral, topical	Ulcers, stomach disorders, swelling	0.27	45	12.15	2.75	0.006	0.6	2.68
*Echinops echinatus* Roxb.	Unt_katara	Asteraceae	PH	Whole plant	Decoction, herbal tea, powder	Oral	Jaundice, liver disorders, flue, hepatitis	0.3	40	12	1.25	0.005	0.55	2.65
*Eclipta prostrata* Lour.	Bhanghra	Asteraceae	AH	Flowers, Roots, leaves	Juice, Powder	Oral	Stomach disorders, blood purification, liver disease	0.25	44	11	3.76	0.008	0.88	3.42
*Emex spinosa* (L.) Campd.	Trkandi Palak	Polygonaceae	AH	Whole plant, leaves	Herbal tea of leaves, decoction	Oral	Fever, pain killer, female disorders	0.22	77	17.11	1.125	0.004	0.49	1.83
*Eragrostis cilianensis* (All.) Vignolo ex Janch.	Candy grass	Poaceae	AG	Whole plant and leaves	Fresh juice, dried powder	Oral, topical	Cough and kidney stones	0.3	70	21	1.334	0.005	0.55	2.65
*Erigeron bonariensis* L.	Beili	Asteraceae	PH	Fruit, leaves, flowers	Herbal tea, infusion	Oral, topical	Diarrhea, stop bleeding, cough, and hepatitis	0.2	42	10.6	3.52	0.008	0.82	2.62
*Erigeron canadensis* L.	Aspi grass	Asteraceae	AH	Whole plant, leaves	Leaves, decoctions, whole plant infusion	Oral	Production and purification of blood hemorrhages	0.26	47	12.22	3.52	0.008	0.82	3.42
*Euphorbia chamaesyce* L.	Dodhak	Euphorbiaceae	AH	Whole plant	Decoctions, powder, paste	Oral, topical	Skin diseases, cough	0.23	46	10.58	2.82	0.007	0.71	2.73
*Euphorbia helioscopia* L.	Dadar boti	Euphorbiaceae	AH	Whole plant	Herbal tea, powder	Oral	Cough, constipation, cancer	0.22	78	17.33	0.6	0.004	0.49	1.83
*Euphorbia hirta* L.	Dhudak booti	Euphorbiaceae	AH	Stem, leaves, seeds	Juice, powder, decoction	Oral	Asthma, cough, worm infestations in children	0.27	45	12.2	2.58	0.006	0.6	2.65
*Farsetia hamiltonii* R.Br.	Fareed-buti	Brassicaceae	PS	Seeds, leaves	Powder, decoction	Oral	Stomach disorders, anti-inflammatory, diabetes, diarrhea	0.27	45	12.2	2.58	0.006	0.6	2.65
*Ficus benghalensis* L.	Bohar	Moraceae	PT	Stem, roots, leaves, flowers	Powder, juice	Oral	Wound healing, gonorrhea, diarrhea	0.27	30	8.1	1.25	0.005	0.55	2.65
*Ficus populifolia* Vahl	Peepal	Moraceae	PT	Fruit, bark, seeds	Powder, paste	Oral, topical	Vomiting, pain relief, diabetes	0.3	50	15	2.5	0.005	0.55	2.65
*Frankenia pulverulenta* L.	Khareya	Frankeniaceae	AH	Flowers, stem, leaves	Decoction, paste	Oral, massage	Skin diseases, analgesic	0.27	45	12.2	2.58	0.006	0.6	2.65
*Fumaria indica* (Hausskn.) Pugsley	Shathara	Papaveraceae	AS	Leaves, whole plant	Powder, decoction	Oral	Fever, liver disease, hypertension	0.18	61	11.07	2.75	0.006	0.6	1.89
* Gamochaeta pensylvanica * (Willd.) Cabrera		Astraceae	AH	Stem, leaves	Herbal tea, decoction	Oral	Digestive disorders, diarrhea, diabetes	0.19	43	8.17	4	0.008	0.88	3.61
*Gisekia pharnaceoides* L.	Balu sag	Aizoaceae	AH	Whole plant	Decoction, powder, and infusion	Oral, topical	Constipation, pain relief, and sexual diseases	0.42	43	18.42	0.467	0.003	0.38	2.57
*Glinus lotoides* L.	Gandi booti	Molluginaceae	AH	Whole plant	Juice, decoction, paste	Oral, topical	Anti-inflammatory, ulcers, wound healing	0.33	40	13.3	1.05	0.004	0.49	2.79
*Heliotropium crispum* Desf.	Kaali lani	Boraginaceae	PH	Seeds, leaves, roots, whole plant	Decoction, powder	Oral, topical	Ulcers, kidney diseases	0.23	53	12.3	3.25	0.007	0.71	2.73
*Heliotropium curassavicum* L.	Lani patta	Boraginaceae	PH	Roots, leaves, flowers	Infusion, paste	Oral, topical	Wound healing, skin, hepatitis	0.17	83	14.16	3	0.006	0.66	1.91
*Heliotropium europaeum* L.	Haathi sundi	Boraginaceae	AH	Whole plant, leaves, flowers	Decoction, infusion	Oral, topical	Skin, wound healing, cough	0.27	45	12.27	2.75	0.006	0.6	2.68
*Imperata cylindrica* (L.) P.Beauv.	Khans	Poaceae	PH	Whole plant	Powder, decoction, infusion	Oral	Heart problems, wounds, fever, urinary disorders, diarrhea	0.21	46	9.69	3.5	0.007	0.77	2.76
*Lantana camara* L.	Pit syapa	Verbenaceae	PS	Whole plant	Decoction, paste, infusion	Oral, topical	Jaundice, Stomach disorders, joint pain, toothache	0.45	36	16.3	1.29	0.006	0.6	4.32
*Lasiurus scindicus* Henrard	Ghorka	Poaceae	PH	Whole plant	Paste, powder, and decoction	Oral, topical	Cough and skin diseases	0.44	44	19.56	1.2	0.004	0.66	3.45
*Launaea mucronata* Muschl.	Desert Grass	Asteraceae	PH	Roots, leaves, stem	Powder, decoction	Oral	Stomach disorders, liver diseases	0.22	56	12.4	0.52	0.004	0.49	3.42
* Launaea nudicaulis * (L.) Hook.f.	Bathal	Solanaceae	AH	Leaves, seeds, stem	Leave infusion, essential oil	Oral, massage, topical	Arthritis, skin disorders, constipation	0.25	62	15.5	0.47	0.004	0.49	2.02
*Lepidium didymum* L.	Afsanteen	Brassicaceae	AH	Leaves, flowers	Decoction, powder	Oral, topical	Blood purifier, liver disorder, stopwound bleeding	0.25	42	10.5	3.2	0.006	0.44	2.05
*Leptadenia pyrotechnica* (Forssk.) Decne.	Khipp	Apocynaceae	PS	Whole plant, seeds, flowers, roots	Paste, decoction	Oral, massage	Cancer, fever, jaundice, constipation, diabetes	0.4	30	12	2.35	0.005	0.66	2.71
*Leptochloa fusca* Kunth	Kallar grass	Poaceae	PG	Leaves, whole plant	Dried powder, herbal tea, paste	Oral, massage	Skin disorders, liver disorders, arthritis, wound healing	0.33	42	14	2.82	0.006	0.55	3.47
*Leucaena leucocephala* (Lam.) de Wit	Jungli shirin	Fabaceae	PT	Seeds, leaves	Dried powder, herbal tea, juice	Oral	Digestive disorders, diabetes, female disorders	0.33	33	10.98	3.75	0.008	0.82	4.38
*Lolium perenne* L.	---	Poaceae	PG	Seeds, leaves, whole plant	Infusion, paste	Oral, massage	Diarrhea, cancer	0.3	50	15	2.67	0.005	0.55	2.65
*Lysimachia arvensis* (L.) U.Manns & Anderb.	Billi buti	Primulaceae	AH	Leaves, flowers, whole plant	Powder, juice	Oral	Fever, liver disease, cancer, stomach disorders	0.25	37	9.25	4	0.008	0.88	3.61
*Malcolmia africana* (L.) W.T.Aiton	Jangle surmee	Brassicaceae	AH	Flowers, leaves, seeds	Powder, paste	Oral, topical	Diabetes, cardiac diseases	0.2	80	16	1.25	0.005	0.55	1.86
*Malva sylvestris* L.	Khubazi	Malvaceae	PH	Leaves, roots	Decoction, paste	Oral	Wound healing, cough, ulcers	0.2	80	16	2.5	0.005	0.55	1.86
*Mazus pumilus* (Burm.f.) Steenis	---	Mazaceae	AH	Whole plant	Decoction, powder	Oral	Fever, sexual disorders	0.3	40	12	2.5	0.005	0.55	2.65
*Medicago polymorpha* L.	Maina	Fabaceae	AH	Leaves, fruit, stem	Powder, infusion	Oral	Liver disorders, hepatitis	0.2	60	12	1.17	0.005	0.55	2
*Melia azedarach* L.	Bakain	Meliaceae	PT	Seeds, leaves, bark, roots	Decoction, juice	Oral	Ulcers, blood purification, skin diseases, rheumatism	0.2	50	10	2.35	0.005	0.55	1.89
*Melilotus albus* Medik.	Jangle sainjee	Fabaceae	AH	Seeds, leaves	Infusion, juice	Oral	Laxative, anti-inflammatory, eye diseases	0.22	66	14.52	0.562	0.006	0.66	3.5
* Melilotus indicus * (L.) All.	Sainji	Fabaceae	PH	Whole plant, seeds	Paste, dried powder, infusion	Oral, topical	Diuretic skin disorders, wound healing, digestive disorders	0.27	45	12.27	1.466	0.004	0.6	2.62
*Melilotus officinalis* Pall.	Sinji	Fabaceae	BH	Flowers, seeds, leaves	Powder	Oral	Vomiting, osteoporosis	0.33	41	13.75	3	0.006	0.49	2.68
*Momordica charantia* L.	Jangli Karela	Cucurbitaceae	AH	Fruit	Decoction, raw	Oral, topical	Diabetes, skin diseases, jaundice, cholesterol, anti-inflammatory	0.36	45	16.36	2.75	0.006	0.6	3.47
*Nerium oleander* L.	Kaner	Apocynaceae	PS	Leaves, flowers	Powder, decoction	Oral	Ulcers, cough, epilepsy	0.35	36	12.8	3.29	0.007	0.77	2.24
*Nicotiana plumbaginifolia* Viv.	Jangle tambaku	Solanaceae	AH	Whole plant, leaves, fruit, flowers	Juice, paste	Oral, topical	Anti-inflammatory, rheumatism, snake bites, wound healing	0.3	40	12	2.5	0.005	0.55	3.48
*Oligomeris linifolia* J.F.Macbr.	Shootk	Resedaceae	AH	Leaves, fruit, whole plant	Powder, decoction	Oral	Fever, jaundice, diarrhea	0.33	33	10.98	3	0.006	0.66	3.5
*Oxalis corniculata* L.	Khati methi booti	Oxalidaceae	PH	Whole plant, leaves, roots, flowers	Decoction, infusion	Oral	Diuretic, diabetes, stomach diseases, fever	0.38	50	19	0.5	0.004	0.44	2.6
* Panicum antidotale * Retz.	Bansi grass	Poaceae	PG	Seeds, stem, leaves	Decoction, herbal tea	Oral	Production and purification of blood, cough, arthritis	0.28	42	11.99	3.5	0.007	0.77	3.56
*Parkinsonia aculeata* L.	Walaiti kikar	Fabaceae	PT	Whole plant, leaves, stem, fruit	Decoction, juice	Oral	Diabetes, anti-inflammatory, pain	0.3	40	12	2.67	0.005	0.55	2.65
*Parthenium hysterophorus* L.	Gajar-ghas	Asteraceae	AH	Leaves, seed, flowers, roots	Powder, decoction, fresh juice	Oral	Diarrhea, diabetes, back pain	0.33	44	14.67	1.2	0.004	0.55	2.65
*Peganum harmala* L.	Harmal	Zygophyllaceae	PH	Seeds, root, bark	Decoction, infusion, paste	Oral, massage	Diabetes, skin diseases, asthma	0.27	36	9.72	2.75	0.006	0.49	2.62
*Persicaria barbata* (L.) Hara	Jor booti	Polygonaceae	AH	Whole plant, root, leaves, seeds	Powder, infusion	Oral, massage	Wound healing, rheumatism, ulcers, astringent	0.44	44	19.5	1.05	0.004	0.55	2.65
*Persicaria glabra* (Willd.) M.Gómez	Booti, gandi booti	Polygonaceae	AH	Whole plant	Decoction, infusion, paste	Oral, topical	Diuretic, pain, cancer	0.27	40	10.8	1.875	0.008	0.49	3.58
* Persicaria lapathifolia * (L.) Delarbre	Gandi buti	Polygonaceae	AH	Whole plant, leaves	Fresh plant juice, decoction, dried powder	Oral	Fever, digestive disorders, dysentery	0.3	50	15	2.5	0.005	0.82	3.58
*Persicaria maculosa* Gray	Ochi	Polygonaceae	AS	Whole plant	Powder, juice, paste	Oral, topical	Anti-inflammatory, constipation, wound healing	0.2	46	9.33	3.75	0.008	0.82	2.79
* Phalaris minor * Retz.	Dumbi siti grass	Poaceae	AH	Seeds, leaves	Leave infusion, herbal tea, fruit juice	Oral	Diuretic, cough, dysentery	0.3	40	12	2.5	0.005	0.55	3.45
*Phoenix dactylifera* L.	Khajoor	Arecaceae	PT	Fruit, leaves	Raw, decoction	Oral	Constipation, throat infections, sexual disorders, toothache, asthma	0.3	40	12	1.334	0.005	0.55	2.65
*Phyla nodiflora* (L.) Greene	Bukkan_boti	Verbenaceae	PH	Whole plant	Extract	Oral, topical	Skin diseases, cough, pain relief	0.45	36	16.3	2.58	0.006	0.6	4.27
*Physalis peruviana* L.	Kakanj	Solanaceae	PS	Fruit, leaves	Fresh fruit juice, dried fruit powder	Oral	Constipation, kidney stones, liver disorders	0.3	40	12	2.35	0.005	0.55	2.68
*Poa annua* L.	Grass	Poaceae	BH	Whole plant	Powder, juice, herbal tea	Oral	Liver disease, diuretic	0.2	47	9.4	4	0.008	0.82	2.79
*Polygonum plebeium* R.Br.	Charri hatha	Polygonaceae	AH	Whole plant	Decoction, powder	Oral	Asthma, diarrhea, sexual diseases, vomiting, blood purification	0.22	55	12.1	0.5625	0.004	0.49	2.62
*Polypogon monspeliensis* (L.) Desf.	Khuth ponch	Poaceae	AH	Leaves, root, whole plant	Decoction, powder, Paste	Oral, topical	Fever, rheumatism, wound healing	0.5	40	20	2.35	0.005	0.55	4.27
*Pontederia crassipes* Mart.	Jal kudi	Pontenderaceae	PH	Stem, Leaves, root	Juice, infusion	Oral	Cancer and skin diseases	0.21	42	8.82	3.5	0.007	0.77	2.76
*Portulaca oleracea* L.	Lonak	Portulacaceae	PH	Root, leaves, whole plant	Decoction, paste, powder	Oral, topical	Blood purifier Skin diseases, jaundice	0.23	38	8.74	1.625	0.007	0.71	2.73
* Prosopis cineraria * (L.) Druce	Jand	Fabaceae	PT	Bark, leaves, seeds	Paste, powder, juice	Oral, topical	Arthritis, cough, asthma	0.21	46	9.66	3.75	0.008	0.82	2.79
*Prosopis Juliflora* (Sw.) DC.	Jangli_keekar	Fabaceae	PT	Leaves, stem	Decoction, powder	Oral, Topical	Skin diseases, cancer	0.27	45	12.27	2.92	0.006	0.6	2.68
*Ranunculus sceleratus* L.	Peeli_boti	Ranunculaceae	PH	Leaves	Decoction	Oral, topical	Pain relief and blood purification	0.2	60	12	2.35	0.005	0.55	1.89
*Rhynchosia minima* (L.) DC.	Tin pinda	Fabaceae	PH	Whole plant	Paste, powder	Oral, topical	Asthma, swelling, wound healing	0.2	60	12	2.35	0.005	0.55	1.91
*Ricinus communis* L.	Arind	Euphorbiaceae	PS	Seeds, Leaves, stem	Infusion, powder	Oral	Jaundice, rheumatism, constipation, eye diseases	0.27	36	9.72	0.6875	0.006	0.6	3.47
*Ridolfia segetum* Moris	Soay	Apiaceae	AH	Seeds and leaves	Decoction and herbal tea	Oral	Eye diseases and stomach ulcers	0.33	44	14.52	0.56	0.004	0.49	1.83
*Rorippa sylvestris *(L.) Besser	-	Brasicaceae	PH	Flower, leaves	Decoction, powder	Oral, topical	Wound healing, skin disorders, urinary diseases, kidney stone	0.36	36	12.96	2.75	0.006	0.6	2.68
*Rumex crispus* L.	Khaar_palak	Polygonaceae	PH	Leaves, fruit	Herbal tea, decoction, infusion	Oral	Used for liver disorder, gastrointestinal diseases	0.57	57	32.57	0.467	0.003	0.38	3.36
*Rumex obtusifolius* L.	Jungli palak	Polygonaceae	PH	Leaves, whole plant	Powder, juice, decoction	Oral	Asthma, cough, arthritis	0.2	60	12	2.35	0.005	0.55	1.91
* Saccharum bengalense * Retz.	Surkanda	Poaceae	PS	Roots, leaves	Leaf juice, powder, decoction	Oral, topical	Skin disorders, fever, constipation, wound healing	0.3	36	10.8	2.75	0.006	0.6	1.89
*Saccharum bengalense* Retz.		Poaceae	PG	Roots, leaves, whole plant	Decoction, powder	Oral	Typhoid, anti-inflammatory	0.33	25	8.33	2.35	0.006	0.66	3.5
*Salsola imbricata* Forssk.	Larha	Amaranthaceae	PS	Whole plant	Decoction, Powder	Oral	Constipation, influenza, and gastrointestinal disease	0.22	55	12.1	3.2	0.004	0.49	2.62
*Salvadora oleoides* Decne.	Wan	Salvadoraceae	PT	Leaves, bark, whole plant	Decoction, paste, herbal tea	Oral, topical	Analgesic, cough, rheumatism, skin diseases	0.21	50	10.5	1.125	0.007	0.77	3.58
*Senna occidentalis* (L.) Link	Kasondi	Fabaceae	PS	Whole plant	Decoction, powder, and paste	Oral, topical	Skin diseases, joint pain, and constipation	0.36	23	8.3	3.5	0.006	0.6	3.47
*Setaria viridis* (L.) P.Beauv.	Sitti_ghaas	Poaceae	AH	Seeds	Powder and infusion	Oral	Lung infections, fever, and constipation	0.25	42	10.5	3.5	0.006	0.66	2.65
*Silene conoidea* L.	Chota takla	Caryophyllaceae	AH	Leaves, bark, stem	Decoction, paste	Oral, topical	Cancer, pain, diabetes, skin diseases	0.18	44	8.25	2.82	0.008	0.88	2.62
*Silybum marianum* (L.) Gaertn.	Ount_katara	Asteraceae	PH	Flowers, leaves, whole plant	Decoction, powder	Oral	Liver disease, jaundice	0.3	30	9.22	3.76	0.007	0.71	3.53
*Sisymbrium irio* L.	jungli_sursoon	Brassicaceae	PH	Leaves, Flowers, roots	Juice, powder, paste	Oral, topical	Liver disease, cough, and wound healing	0.2	60	12	3.25	0.005	0.55	2.65
*Solanum esuriale* Lindl.	Kantakari	Solanaceae	PH	Whole plant, leaves, root, bark	Juice, paste, infusion	Oral, topical	Liver disorders, cough, asthma, fever	0.27	45	12.2	3.05	0.006	0.6	2.68
*Solanum nigrum* Acerbi ex Dunal	Mako	Solanaceae	AH	Whole plant	Decoction, juice, infusion	Oral, topical	Liver disorders, joint pain, diabetes	0.33	44	14.52	1.25	0.004	0.49	1.83
*Solanum virginianum* L.	Choti_kateri/kanderi	Solanaceae	PH	Leaves, fruit, seeds, and flowers	Powder, paste, and infusion	Oral, topical	Asthma, diabetes, and joint pain	0.23	46	10.6	1.125	0.007	0.71	2.68
*Sonchus asper* (L.) Hill	Didhi	Asteraceae	PH	Whole plant	Decoction, paste, powder	Oral, topical	Used for constipation, wounds, cough, fever	0.28	28	7.99	2.58	0.007	0.77	3.56
*Sonchus oleraceus* f. hydrophilus (Boulos) J.Kost.	Smooth sowthistle	Asteraceae	AH	Leaves, roots, whole plant	Juice, powder	Oral	Anti-inflammatory, gastrointestinal disorders, anti-tumor	0.4	40	16	3.5	0.005	0.55	3.45
*Sonchus oleraceus* L.	Smooth sowthistle	Asteraceae	AH	Leaves, roots, whole plant	Juice, powder	Oral	Anti-inflammatory, gastrointestinal disorders, anti-tumor	0.25	41	10.25	3	0.006	0.66	3.5
* Spergula arvensis * L.	Jungli dhania	Caryophyllaceae	AH	Leaves and seeds	Fresh infusion of leaves	Oral	Kidney stones and diuretic	0.28	71	20.29	0.466	0.003	0.38	1.78
*Stellaria media* (L.) Vill.	Speengulay	Caryophyllaceae	PH	Flowers, leaves, roots, whole plant	Herbal tea, powder, paste	Oral, topical	Anti-inflammatory, asthma, swelling	0.33	44	14.52	0.562	0.004	0.49	1.83
* Suaeda nigra * (Raf.) J.F.Macbr.	Laani	Chenopodiaceae	PH	Leaves, stem	Infusion, juice, paste	Oral, topical	Constipation, eye diseases, skin disorders	0.3	40	12	1.334	0.005	0.55	2.65
*Suaeda vermiculata* Forssk. ex J.F.Gmel.	Laani	Amaranthaceae	PS	Whole plant	Powder, herbal tea	Oral	Kidney diseases and skin and eye problems	0.23	53	12.38	3.25	0.007	0.71	2.73
*Tamarix aphylla* (L.) H.Karst.	Frash/Ukan	Tamaricaceae	PT	Bark, leaves, whole plant	Herbal tea, powder	Oral	Anti-inflammatory, rheumatism, hepatitis	0.3	40	12	1.25	0.005	0.55	1.86
*Trianthema portulacastrum* L.	Itsit	Aizoaceae	PH	Roots, leaves, whole plant	Powder, infusion	Oral	Blood purifier, jaundice, asthma, cough	0.27	40	10.8	1.875	0.008	0.82	3.58
*Tribulus terrestris* L.	Bakhra	Zygophyllaceae	AH	Flowers, fruits	Juice, decoction, dried powder	Oral	Cough, urinary diseases, kidney stones	0.23	54	12.46	3.468	0.007	0.71	2.73
*Trifolium repens* L.		Fabaceae	PH	Flowers, roots, leaves	Infusion, powder	Oral	Fever, jaundice cough	0.3	40	12	2.75	0.005	0.55	2.65
*Trifolium resupinatum* L.	Shatala	Fabaceae	AH	Whole plant, flowers	Juice, herbal tea	Oral	Cancer, ulcers, skin disorders, cough	0.36	36	13.17	2.5	0.006	0.6	3.47
*Vachellia nilotica* (L.) P.J.H.Hurter & Mabb.	kikar	Fabaceae	PT	Seeds, leaves	Dried powder, fresh paste	Oral	Diabetes, cough, fever, dysentery	0.33	50	9.5	3.2	0.006	0.66	3.5
* Verbascum virgatum * Stokes	-	Scrophulariaceae	PH	Flowers and leaves	Decoction and infusion	Oral	Cough, asthma, and diuretic	0.43	57	24.43	0.467	0.003	0.38	2.57
*Veronica anagallis-aquatica* L.	Pani wali boti	Plantaginaceae	PH	Leaves, roots	Decoction, powder, paste	Oral, topical	Urinary disorders and blood purification, appetizer	0.33	44	14.6	0.52	0.004	0.49	2.82
*Vicia hirsuta *(L.) Gray	Jungli matri	Fabaceae	AH	Leaves and seeds	Herbal tea, powder	Oral	Pain killer, anti-inflammatory	0.22	67	14.89	1.2	0.004	0.49	1.83
*Vicia sativa* L.	Mattri	Fabaceae	BH	Seeds, leaves, whole plant	Herbal tea, decoction	Oral	Anti-inflammatory, bronchitis, muscle cramps	0.27	45	12.15	1.375	0.006	0.6	2.68
*Vincetoxicum spirale* (Forssk.) D.Z.Li	Hiran boti	Asclepiadaceae	PH	Whole plant	Herbal tea, powder	Oral	Wounds, stop bleeding, stomach disorders, anti-inflammatory	0.3	50	15	2.5	0.005	0.6	2.68
*Withania somnifera* (L.) Dunal	AK-san	Solanaceae	PS	Bark, leaves, root, fruits	Infusion, paste, powder	Oral, topical	Asthma, insomnia, swelling	0.19	43	8.17	2	0.008	0.88	3.61
*Xanthium strumarium* L.	Leedha	Asteraceae	AH	Whole plant	Paste, decoction, infusion	Oral, topical	Fever, flue, animal bites, pain reliever	0.26	40	10.6	3.52	0.008	0.82	3.45
*Zaleya pentandra* (L.) C. Jeffrey	Iit sit	Aizoaceae	PH	Whole plants, leaves, roots	Juice, powder	Oral	Cough, kidney stones, digestive disorders	0.21	50	10.71	3.73	0.007	0.77	2.76
*Ziziphus jujuba* Mill.	Beri	Rhamnaceae	PT	Fruit, leaves, seeds	Decoction, infusion, powder	Oral	Constipation, cough, asthma	0.3	50	15	2.67	0.005	0.49	3.42
* Ziziphus nummularia * (Burm.f.) Wight & Arn.	Paindo beri	Rhamnaceae	PT	Fruits, seeds	Fresh fruit juice, dried fruit powder	Oral	Diabetes, skin disorders, anti-inflammatory	0.3	50	15	2.67	0.005	0.55	2.65
*Ziziphus spina-christi* (L.) Willd.	Choti-berry	Rhamnaceae	PT	Bark, seeds, leaves fruits, whole plant	Paste, powder	Oral, topical	Skin diseases, jaundice, eye diseases, hepatitis	0.44	33	14.52	1.125	0.004	0.55	2.65
*Zygophyllum creticum* (L.) Christenh. & Byng	Dhamasa Boti	Zygophyllaceae	PH	Whole plant	Decoction, powder, infusion	Oral	Jaundice, blood purification, asthma	0.16	67	11.1	3.25	0.006	0.66	1.86

BN—botanical name, LN—local name, LF—life form, PU—part used, Rec—recipe, App—mode of application, UV—use value, RFC—relative frequency citation, RI—relative importance, CSI—cultural significance index, FI—frequency index, ROP—rank order priority, FL—fidelity level, AH—annual herb, PH—perennial herb, AS—annual shrub, PS—perennial shrub, PT—perennial tree, AG—annual grass, PG—perennial grass.

**Table 3:** Informant consensus factor (ICF) and popular therapeutic use value (POPUT).

Diseases	ICF	POPUT	Diseases	ICF	POPUT
Urinary Infections	0.81	0.012	Headache	1.00	0.001
Analgesic	0.93	0.008	Heart diseases	1.00	0.006
Animal Bite	0.8	0.006	Hemorrhages	1.00	0.003
Anti-inflammatory	0.87	0.046	Hepatitis	0.73	0.014
Anti-microbial	1.00	0.003	Hypertension	0.88	0.005
Arthritis	0.8	0.022	Insomnia	1.00	0.002
Asthma	0.76	0.06	Jaundice	0.75	0.036
Back pain	1.00	0.002	Joint pain	0.81	0.009
Blood pressure	1.00	0.002	Kidney disorders	0.84	0.031
Bronchitis	0.86	0.004	Laxative	0.83	0.004
Cancer	0.76	0.019	Liver disorders	0.79	0.039
Cholesterol	0.75	0.002	Lung infection	0.87	0.008
Constipation	0.76	0.052	Muscle cramps	1.00	0.002
Cough	0.27	0.077	Osteoporosis	1.00	0.002
Diabetes	0.68	0.039	Pain killer	0.78	0.033
Diarrhea	0.72	0.031	Paralysis	1.00	0.003
Digestive disorders	0.74	0.019	Piles	0.80	0.003
Diuretic	0.76	0.035	Production and purification of blood	0.83	0.032
Dysentery	0.75	0.005	Rheumatism	0.76	0.018
Earache	1.00	0.002	Sexual disorders	0.76	0.012
Appetizer	1.00	0.002	Skin disorders	0.26	0.088
Epilepsy	1.00	0.002	Snake bite	1.00	0.002
Eye diseases	0.31	0.018	Stomach disorder	0.76	0.032
Female disorders	0.6	0.003	Stop bleeding	0.50	0.002
Fever	0.79	0.066	Swelling	0.79	0.011
Flu	0.88	0.013	Throat infection	0.67	0.002
Gargles	1.00	0.001	Toothache	0.78	0.006
Gastrointestinal disorders	0.73	0.008	Anti-tumor	1.00	0.002
Gonorrhea	1.00	0.003	Typhoid	1.00	0.002
Hair treatment	1.00	0.002	Ulcer	0.70	0.022
Wound healing	0.73	0.05	Vomiting	0.81	0.012
Worm infestation in children	1.00	0.002	-	-	-

**Table 4:** Family use value (FUV) and family importance value (FIV).

Plant Families	No. of Species	FIV	FUV
Aizoaceae	3	1.89	0.27
Amaranthaceae	9	5.69	0.29
Apiaceae	1	0.63	0.27
Apocynaceae	4	2.53	0.31
Arecaceae	1	0.63	0.45
Asclepiadaceae	1	0.63	0.44
Asphodelaceae	1	0.63	0.23
Asteraceae	14	8.86	0.27
Boraginaceae	3	1.89	0.25
Brassicaceae	5	3.16	0.35
Caesalpiniaceae	1	0.63	0.36
Capparaceae	2	1.26	0.32
Capparidaceae	1	0.63	0.27
Caryophyllaceae	3	1.89	0.3
Chenopodiaceae	3	1.89	0.32
Cleomaceae	1	0.63	0.28
Convolvulaceae	2	1.26	0.22
Cucurbitaceae	3	1.89	0.29
Cyperaceae	2	1.26	0.26
Euphorbiaceae	4	2.53	0.29
Fabaceae	21	13.29	0.18
Frankeniaceae	1	0.63	0.22
Gentianaceae	1	0.63	0.37
Malvaceae	2	1.26	0.2
Mazaceae	1	0.63	0.35
Meliaceae	2	1.26	0.3
Molluginaceae	1	0.63	0.23
Moraceae	2	1.26	0.27
Nyctaginaceae	2	1.26	0.32
Orbanchaceae	1	0.63	0.28
Oxalidaceae	1	0.63	0.28
Papaveraceae	1	0.63	0.19
Plantaginaceae	1	0.63	0.33
Poaceae	21	13.29	0.29
Polygonaceae	9	5.69	0.3
Pontenderaceae	1	0.63	0.22
Portulacaceae	1	0.63	0.21
Primulaceae	1	0.63	0.25
Pteridaceae	1	0.63	0.25
Ranunculaceae	1	0.63	0.2
Resedaceae	1	0.63	0.38
Rhamnaceae	3	1.89	0.35
Salvadoraceae	1	0.63	0.36
Sapindaceae	1	0.63	0.3
Scrophulariaceae	1	0.63	0.43
Solanaceae	9	5.69	0.25
Tamaricaceae	1	0.63	0.3
Verbenaceae	2	1.26	0.32
Zygophyllaceae	3	1.89	0.27

**Table 5:** Preference ranking (PR) of medicinal plants used to cure various ailments in the study area.

Botanical Names	Illness	Respondents											
		R1	R2	R3	R4	R5	R6	R7	R8	R9	R10	Score	Rank
*Melia azedarach* L.	Skin diseases	3	2	3	2	3	2	3	3	1	3	25	1st
*Peganum harmala* L.		3	2	3	3	2	2	2	3	2	2	24	2nd
*Cymbopogon jwarancusa* (Jones) Schult		3	2	2	2	3	1	3	3	3	0	22	3rd
*Momordica charentia* L.		2	3	3	3	2	2	3	3	0	0	21	4th
*Senna occidentalis* (L.) Link.		2	2	3	3	3	3	2	3	0	0	21	4th
*Convolvulus arvensis* L.		3	3	3	3	2	2	2	3	0	0	21	4th
*Eichhornia crassipes* Mart.		3	1	3	1	3	1	3	1	2	2	20	5th
*Salvadora oleoides* Decne.		1	1	2	2	2	2	2	2	3	2	19	6th
*Azadirachta indica* A. Juss.		2	2	2	3	3	2	1	3	0	0	18	6th
*Trifolium resupinatum* L.		1	2	2	3	1	3	2	1	1	2	18	6th
*Euphorbia prostrata* Aiton		2	2	2	2	3	2	2	2	0	0	17	7th
*Phyla nodiflora* L. Greene		3	2	2	1	2	1	2	3	0	0	16	8th
*Prosopis Juliflora* (Sw.) DC		2	2	1	1	2	3	3	2	0	0	16	8th
*Heliotropium curassavicum* L.		1	2	2	1	2	1	1	1	3	0	14	9th
*Bauhinia variegata* L.	Fever	3	2	3	2	2	2	3	3	0	0	20	1st
*Achyranthes aspera* L		3	2	2	3	2	3	2	2	0	0	19	2nd
*Setaria viridis* (L.) P		2	3	2	3	3	3	2	1	0	0	19	2nd
*Imperata cylindrica* (L.)		2	2	1	3	2	2	3	3	0	0	18	3rd
*Saccharum bengalensis* Retz.		2	1	3	3	2	2	3	2	0	0	18	3rd
*Mazus pumilus* (Burm. F.)		2	2	3	2	3	2	1	2	0	0	17	4th
*Alhagi maurorum* Medik.		2	1	3	2	3	2	3	1	0	0	17	4th
*Sonchus asper* (L.) Hill.		2	2	2	3	2	2	2	1	0	0	16	5th
*Zaleya pentandra* (L.)		2	1	3	2	2	2	1	3	0	0	16	5th
*Acacia nilotica* (L.)		2	3	3	2	1	1	2	2	0	0	16	5th
*Xanthium strumariam* L.		2	2	2	3	2	1	1	2	0	0	15	6th
*Dactyloctenium aegyptium* L.		1	2	1	1	2	2	3	1	0	0	13	7th
*Phoenix dactylifera* L.	Constipation	3	3	3	3	3	3	2	3	0	0	23	1st
*Convolvulus arvensis* L.		3	3	3	3	2	2	2	3	0	0	21	2nd
*Senna occidentalis* (L.) Link		2	2	3	3	3	3	2	3	0	0	21	2nd
*Setaria viridis* (L.) P		2	3	2	3	3	3	2	1	0	0	19	3rd
*Saccharum bengalensis* Retz.		2	1	3	3	2	2	3	2	0	0	18	4th
*Rumex crispus* L.		2	2	2	3	2	2	2	3	0	0	18	4th
*Sonchus asper* (L.) Hill.		2	2	2	3	2	2	2	1	0	0	16	5th
*Euphorbia heliscopia* L.		2	2	2	1	2	2	3	2	0	0	16	5th
*Zizipus jujuba* Mill.		2	3	2	2	2	3	1	1	0	0	16	5th
*Sueda nigra* (Rafinesque) J.		2	2	1	3	2	1	2	2	0	0	15	6th
*Gisekia pharnaceoides* L.		1	2	2	2	2	1	3	2	0	0	15	6th
*Chenopodium murale* L.		2	2	1	1	2	1	1	2	0	0	12	7th
*Farsetia hamiltonii* Royle.	Inflammation	2	3	3	2	3	2	3	3	0	0	21	1st
*Parkinosnia aculeata* L.		2	3	2	3	2	2	2	3	1	0	20	2nd
*Tamarix aphylla* (L.) Karst.		3	2	3	2	3	2	3	2	0	0	20	2nd
*Persicaria maculosa* S. F. Gay		2	2	3	2	2	3	4	2	0	0	20	2nd
*Cymbopogon jwarancusa* (Jones) Schult		2	3	1	2	2	1	3	2	3	0	19	3rd
*Sonchus oleraceus* L.		2	2	3	2	2	3	3	2	0	0	19	3rd
*Boerhavia procumbens* Banks ex Roxb.		2	3	2	2	3	3	2	2	0	0	19	3rd
*Vicia sativa* L.		2	3	2	2	2	1	3	3	0	0	18	4th
*Cleome brachycarpa* Vahl.		1	2	2	1	1	2	2	3	3	0	17	5th
*Crotalaria burhia* Buch.-Ham. ex Benth.		2	2	2	2	2	3	2	2	0	0	17	5th
*Glinus lotoides* L.		3	1	2	1	1	2	1	2	3	0	16	6th
*Melilotus alba* Desr.		2	1	2	1	1	3	2	3	0	0	15	7th
*Stellaria media* (L.) Villars		2	2	1	1	3	2	2	2	0	0	15	7th
*Cyperus rotundus* L.		3	2	2	1	1	2	2	1	0	0	14	8th
*Datura alba* L.		1	1	2	3	1	3	1	2	0	0	14	8th
*Saccharum munja* Roxb.		2	1	2	1	2	3	1	1	0	0	13	9th
*Abutilon indicum* (L.) Sweet		2	1	2	1	2	1	2	1	0	0	12	10th
*Convolvulus arvensis* L.	Blood purifying	3	3	3	3	2	2	2	3	0	0	21	1st
*Fagonia cretica* L.		2	2	3	3	2	3	3	3	0	0	21	1st
*Dalbergia sisso* Roxb. Ex. DC.		3	2	2	3	3	3	3	2	0	0	21	1st
*Ranunculus sceleratus* L.		2	2	3	3	2	2	2	2	0	0	18	2nd
*Veronica anagallis-aquatica* L.		3	2	2	2	3	1	2	3	0	0	18	2nd
*Azadirachta indica* A. Juss.		2	2	2	3	3	2	1	3	0	0	18	2nd
*Lepidium didymium* L.		2	2	3	2	2	2	3	2	0	0	18	2nd
*Polygonum plebejum* R.Br.		1	2	2	2	2	1	3	2	0	0	15	3rd
*Salvadora oleoides* Decne.	Cough	2	3	2	3	3	2	2	3	2	2	24	1st
*Capparis spinosa* L.		2	3	2	1	3	2	1	3	2	3	22	2nd
*Calotropis gigantea* L.		3	2	3	2	3	3	1	1	0	2	20	3rd
*Heliotropium europaeum* L.		3	2	1	1	1	3	2	2	2	2	19	4th
*Trifolium repens* L.		2	3	3	2	2	2	2	2	0	0	18	5th
*Cymbopogon jwarancusa* (Jones) Schult		1	2	1	1	2	3	3	3	1	1	18	5th
*Trifolium resupinatum* L.		3	1	1	2	3	2	2	1	1	0	16	6th
*Anchusa arvensis* (L.) Bieb.		1	2	1	3	1	2	3	2	0	0	15	7th
*Malva parviflora* L.		1	2	1	2	1	1	1	2	3	1	15	7th
*Euphorbia hirta* L.		3	1	3	2	2	3	1	2	0	0	14	8th
*Cirsium arvense* (L.) Scop.		3	2	3	2	2	2	3	2	0	0	14	8th
*Rumex obtusifolius* L.		2	2	2	2	3	3	3	2	0	0	14	8th
*Trianthema portulacastrum* L.		2	2	2	3	2	3	3	1	0	0	14	8th
*Nerium oleander* L.		2	3	2	2	3	1	2	3	0	0	13	9th
*Sisymbrium irio* L.		2	2	2	3	2	2	1	1	0	0	13	9th

**Table 6:** Jaccard Index (JI).

Area	SY	TRS	NPSU	NPDU	TSCBA	PPSU	PPDU	JI	Citation
Cholistan desert	2023	123	44	18	62	35.77	14.63	28.31	[1]
Bahawalpur	2014	93	31	24	55	33.33	25.80	28.06	[16]
Yazman	2019	118	45	13	58	38.13	11.01	26.60	[17]
Hafizabad	2017	85	25	14	39	29.41	16.47	19.11	[13]
Bahawalnagar	2020	81	23	12	35	28.39	14.81	17.15	[18]
Sargodha	2012	98	15	16	31	15.30	16.32	13.77	[19]
Okara	2022	126	13	21	34	10.31	16.66	13.6	[20]
Multan	2014	44	9	14	23	20.45	31.81	12.84	[21]
Southern Punjab	2021	58	12	12	24	20.68	20.68	12.5	[22]
District Buner, Khyber Pakhtunkhwa	2022	106	8	13	21	7.54	12.26	8.64	[11]
Gujrat	2014	37	5	4	9	13.51	10.81	4.83	[14]
Iran	2019	36	0	7	7	0	19.44	3.74	[23]
Haripur District, Khyber Pakhtunkhwa	2021	40	1	6	7	2.5	15	3.66	[24]
India	2022	60	1	2	3	1.66	3.33	1.39	[25]
China	2022	121	0	0	0	0	0	0	[26]

SY—study year, TRS—total number of reported species, NPSU—number of plants with similar uses, NPDU—number of plants with dissimilar uses, TSCBA—total number of species common in both areas, PPSU—percentage of plants with similar uses, PPDU—percentage of plants with dissimilar uses, JI—Jaccard index.

## Data Availability

All the data used to support this study are included in the paper.
